# Deep learning-based automatic measurement of the femoral head ossification center in healthy Korean children: development of a novel radiographic growth chart

**DOI:** 10.1007/s00330-025-12263-z

**Published:** 2026-01-13

**Authors:** Byoung-Dai Lee, Ki-Ryum Moon, Jin Young Kim, Mu Sook Lee

**Affiliations:** 1 Department of Computer Science, Graduate School, Kyonggi University, Suwon-si, Republic of Korea; 2 Department of Radiology, Keimyung University Dongsan Hospital, Daegu, Republic of Korea

**Keywords:** Artificial intelligence, Child development, Femur head, Growth charts, Radiography

## Abstract

**Objectives:**

To develop and validate a deep learning (DL)-based algorithm for automated measurement of femoral head ossification center (FHOC) size and establish AI-derived growth charts.

**Materials and methods:**

This retrospective study included 1705 healthy Korean children (mean age, 5.1 ± 3.3 years; 841 females, 864 males) with anteroposterior pelvic radiographs (2018–2024). A three-stage DL algorithm (region-of-interest detection, FHOC segmentation, landmark-based size computation) was used to automatically measure FHOC size. Agreement with radiologist measurements was evaluated using concordance correlation coefficient (CCC), Pearson correlation coefficient (*r*), mean absolute error (MAE), root mean square error (RMSE), and Bland–Altman analyses, supplemented by paired *t*-test and Fisher’s *Z*-test. AI measurements were used to create FHOC growth charts via quantile polynomial regression, with predictive accuracy assessed by adjusted *R*², MAE, and RMSE.

**Results:**

AI-derived FHOC size measurements showed close agreement with radiologist measurements, with mean differences within ±0.5 mm and 95% limits of agreement within ±3 mm in age-stratified analyses, and overall agreement was further supported by high CCC, *r*, and consistently low error metrics. Growth curves based on AI measurements demonstrated strong predictive accuracy (adjusted *R*² = 0.927 for females; 0.934 for males), with low errors across age groups (females: MAE 1.77–2.98 mm, RMSE 2.28–3.54 mm; males: MAE 1.60–3.01 mm, RMSE 2.00–4.10 mm). Reference percentiles (5th–95th) were established, providing standardized FHOC size ranges for clinical application.

**Conclusion:**

Our DL-based approach provides precise and reproducible FHOC size measurement, offering a robust reference for standardized growth assessment and early pediatric hip joint evaluation.

**Key Points:**

***Question****The timing of FHOC appearance is an important radiographic indicator; however, manual measurement is subjective, and studies on age-specific changes remain limited*.

***Findings****A DL-based algorithm achieved high agreement with expert measurements, and age-based regression reliably predicted FHOC size in children*.

***Clinical relevance****AI-derived FHOC growth charts may provide objective, standardized references for pediatric hip joint development, potentially enabling earlier detection of growth abnormalities and improving diagnostic consistency in clinical practice*.

**Graphical Abstract:**

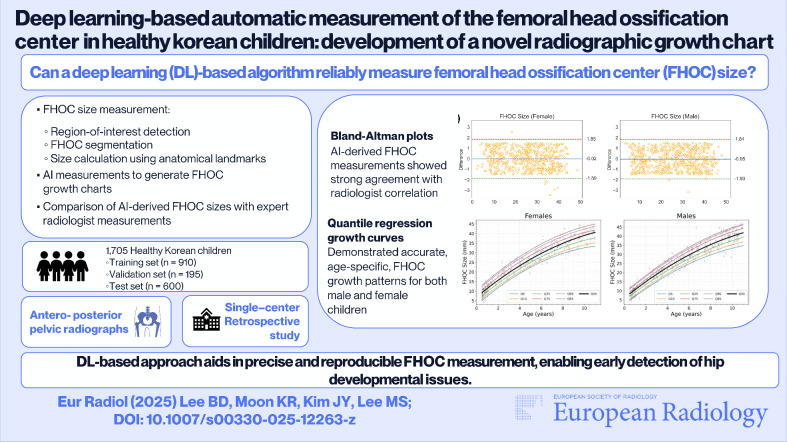

## Introduction

Conventional pelvic radiography is the primary imaging modality for evaluating pediatric hip joints in most routine clinical practices ([1–3]). However, the pediatric hip joint undergoes progressive morphological changes during growth, as ossification centers emerge and mature [4]. Therefore, careful and accurate interpretation requires assessing hip joint morphology, proximal femur shape, and ossification degree in relation to the patient’s developmental stage ([5, 6]). Various radiographic angular and linear measurements of the hip joint are routinely used to diagnose and monitor hip joint abnormalities ([7–11]). Additionally, the appearance of the femoral head ossification center (FHOC), which reportedly appears at 4 months in 50% and 7–8 months in 95%, serves as an essential indicator for assessing conditions such as skeletal dysplasia, endocrinopathies, and developmental dysplasia of the hip (DDH) ([12, 13]).

Despite its clinical importance, previous studies have predominantly focused on the timing and symmetry of FHOC appearance, with limited research addressing age-specific changes in FHOC size from infancy through adolescence [14]. Manual radiographic measurements, including FHOC size, are known from previous studies to be subject to limitations such as inter- and intra-observer variability [9], substantial time requirements for large-scale assessments [15], and reduced reproducibility due to ambiguous anatomical landmarks or variable image quality [16]. These challenges underscore the need for automated, standardized measurement approaches that can mitigate human variability and improve efficiency. Recent advances in artificial intelligence (AI) have demonstrated promising performance in pediatric musculoskeletal imaging, including automated bone age assessment, growth plate detection, and skeletal segmentation ([17–19]). These AI-based methods have shown potential to improve reproducibility, minimize observer dependence, and significantly accelerate measurement workflows. Leveraging similar methodologies for FHOC assessment may overcome the intrinsic limitations of manual measurements, enabling consistent, objective, and scalable evaluations in both clinical and research settings.

Given these considerations, the purpose of this study was to develop a deep learning (DL)-based algorithm to enable accurate and reproducible FHOC size measurement and to establish a radiographic growth chart for healthy children. By leveraging advanced image analysis techniques, this approach aims to enhance the objectivity and efficiency of pediatric hip joint assessments, providing standardized reference values for precise diagnosis and proper follow-up for various pediatric hip joint diseases.

## Materials and methods

### Study participants

This retrospective study was approved by the institutional review board, which waived the requirement for informed and/or parental consent due to the use of de-identified, pre-existing clinical data obtained for routine patient care and the minimal risk to participants. We manually searched the picture archiving and communication system (PACS) at our University Medical Center to identify anteroposterior (AP) pelvic radiographs of healthy Korean children obtained between 2018 and 2024. Healthy children were defined as those with height and body weight between the 3rd and 97th percentiles for age and sex, according to the 2017 Korean National Growth Chart [20], with no history of musculoskeletal or systemic conditions affecting bone development—including chronic illness, endocrinopathy, neuromuscular disease, intrinsic bone disease, metabolic disorders, previous skeletal trauma, or surgery—and no abnormal findings on pelvic radiographs. Additionally, only radiographs demonstrating pelvic rotation and tilt within normal ranges (pelvic rotation index 0.56–1.8 and pelvic tilt index 0.75–1.2 [21]) were included to ensure consistent anatomical positioning. All radiographs were acquired using the EOS imaging system, which provides low-dose radiographs with minimal geometric distortion [22]. Although EOS examinations often include both AP and lateral views, only the 2D AP pelvic radiographs were used in this study.

### DL model training and evaluation

**Fig. 1:**
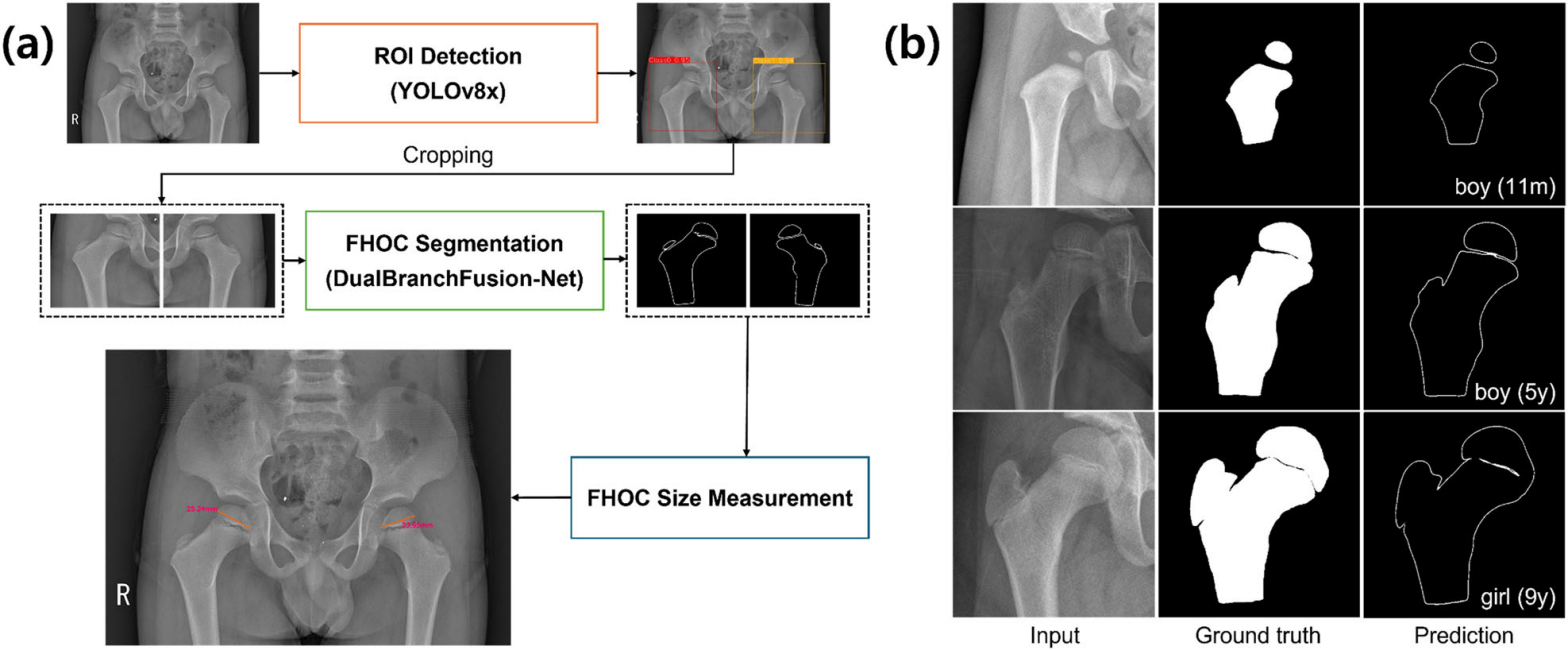
Pipeline of the cascaded DL-based algorithm and representative examples of FHOC segmentation. **a** Step-by-step process of the proposed method, including ROI extraction, FHOC segmentation, and FHOC size measurement. The FHOC size is computed as the maximum transverse diameter between two automatically detected cortical landmarks on the segmented ossification center (see Supplementary Appendix S3 for full algorithm details). **b** Examples of FHOC segmentation results at different stages, illustrating the model’s ability to accurately delineate FHOC boundaries. FHOC, femoral head ossification center; ROI, region of interest

A three-stage cascaded DL-based algorithm was developed to segment the FHOCs from AP pelvic radiographs and automatically measure their size (Fig. 1). In the first stage, a region of interest (ROI) detection network was trained to localize bounding boxes containing the left and right FHOCs. In the second stage, an image segmentation network was trained to segment the FHOC within each detected ROI. In the third stage, a post-processing algorithm detected key landmarks from the segmentation masks, and the FHOC size was then calculated based on these landmarks. All training and testing procedures were performed on a single NVIDIA A100 GPU with 80 GB RAM. Further details on the model architecture, data preprocessing, and training procedures for the object detection and segmentation networks are provided in Supplementary Appendices S1 and S2. Supplementary Appendix S3 presents the landmark detection algorithm. The related code is available at https://github.com/ACSLab123/FHOCAI.

The dataset consisted of 1705 de-identified AP pelvic radiographs from 1705 different children. These radiographs were randomly divided into training (*n* = 910, 53.4%), validation (*n* = 195, 11.4%), and test (*n* = 600, 35.2%) subsets, with a strict patient-level split to prevent participant overlap among the subsets. The test set was further stratified into 24 age- and sex-specific subgroups (12 age groups × 2 sexes), each comprising approximately 25 participants per sex, yielding a balanced sample of 50 bilateral pelvic measurements from left and right hip joint assessments. All model evaluations and statistical analyses were conducted exclusively on this independent test set to ensure unbiased performance assessment and statistical validity. This sampling strategy, consistent with established methodologies in pediatric morphometric research (e.g., Wegener et al [23]), provides balanced subgroup representation and sufficient statistical power for evaluating agreement between AI-derived and radiologist measurements. For DL model development, FHOC bounding boxes and segmentation masks were manually annotated by four medical data annotators trained in musculoskeletal radiograph interpretation, under the supervision of a board-certified radiologist with 22 years of experience. The dataset was divided among the annotators, and all annotations were systematically reviewed and confirmed by the supervising radiologist.

### Measurement of FHOC sizes

The FHOC size was defined as the maximum transverse diameter of the capital femoral epiphysis, measured as the linear distance from the medial margin to the outermost lateral point of the femoral head. This measurement captures the greatest width of the ossification center, a dimension that reflects overall morphological development and correlates closely with hip joint maturation and skeletal growth and has been validated in prior pediatric musculoskeletal studies ([23, 24]). Moreover, it demonstrates sensitivity to growth disturbances in pathological conditions such as DDH and Legg–Calvé–Perthes (LCP) disease ([23, 25, 26]).

To establish reference standards, FHOC sizes in the test set were manually measured by a board-certified radiologist with 22 years of experience using a clinical PACS workstation. The radiologist was blinded to AI-derived measurements during manual assessment to prevent potential bias.

### Evaluation metrics and statistical analysis

The evaluation metrics were selected according to established practices in medical image analysis and tailored to each specific task. All statistical analyses were performed using Python [27], with *p* < 0.05 considered statistically significant.

#### Evaluation metrics

ROI detection was evaluated using mean average precision (mAP), which provides a comprehensive measure of object localization accuracy across confidence thresholds. Segmentation performance was assessed with the Dice similarity coefficient (DSC) [28] and Hausdorff distance (HD) [29], reflecting region overlap and boundary accuracy, respectively. Landmark detection accuracy was evaluated using the mean radial error (MRE) [30] and successful detection rate (SDR) [31], which quantify both mean localization precision and robustness.

#### Agreement and subgroup analysis

Agreement between AI-derived FHOC size measurements and radiologist measurements was assessed using the concordance correlation coefficient (CCC), which evaluates agreement around the line of identity, and Pearson’s correlation coefficient (*r*), which measures linear association irrespective of bias; this was complemented by mean absolute error (MAE) and root mean square error (RMSE) as absolute and squared difference measures. Bland–Altman analysis was used to characterize measurement bias and 95% limits of agreement (LoA), supporting assessment of clinical interchangeability. A supplementary paired *t*-test was performed to compare mean measurements between the two methods, with results provided for completeness, while agreement metrics were used as the primary evaluation tools. To evaluate subgroup differences, Fisher’s *Z*-tests [30] were used to statistically compare correlation coefficients between sex-based (male vs female) and laterality-based (left vs right) groups. Laterality-based Bland–Altman analyses were also performed to assess measurement consistency between left and right hips

#### Predictive modeling

For predictive modeling, quantile regression with polynomial growth curves was performed on AI-derived FHOC size measurements, with a monotonic non-decreasing constraint to ensure biologically plausible growth trajectories. Percentile curves (5th, 10th, 25th, 50th, 75th, 90th, and 95th) were generated, and model performance was evaluated using adjusted *R*², MAE, and RMSE to reflect both explanatory power and prediction accuracy.

## Results

### Study participants

**Fig. 2:**
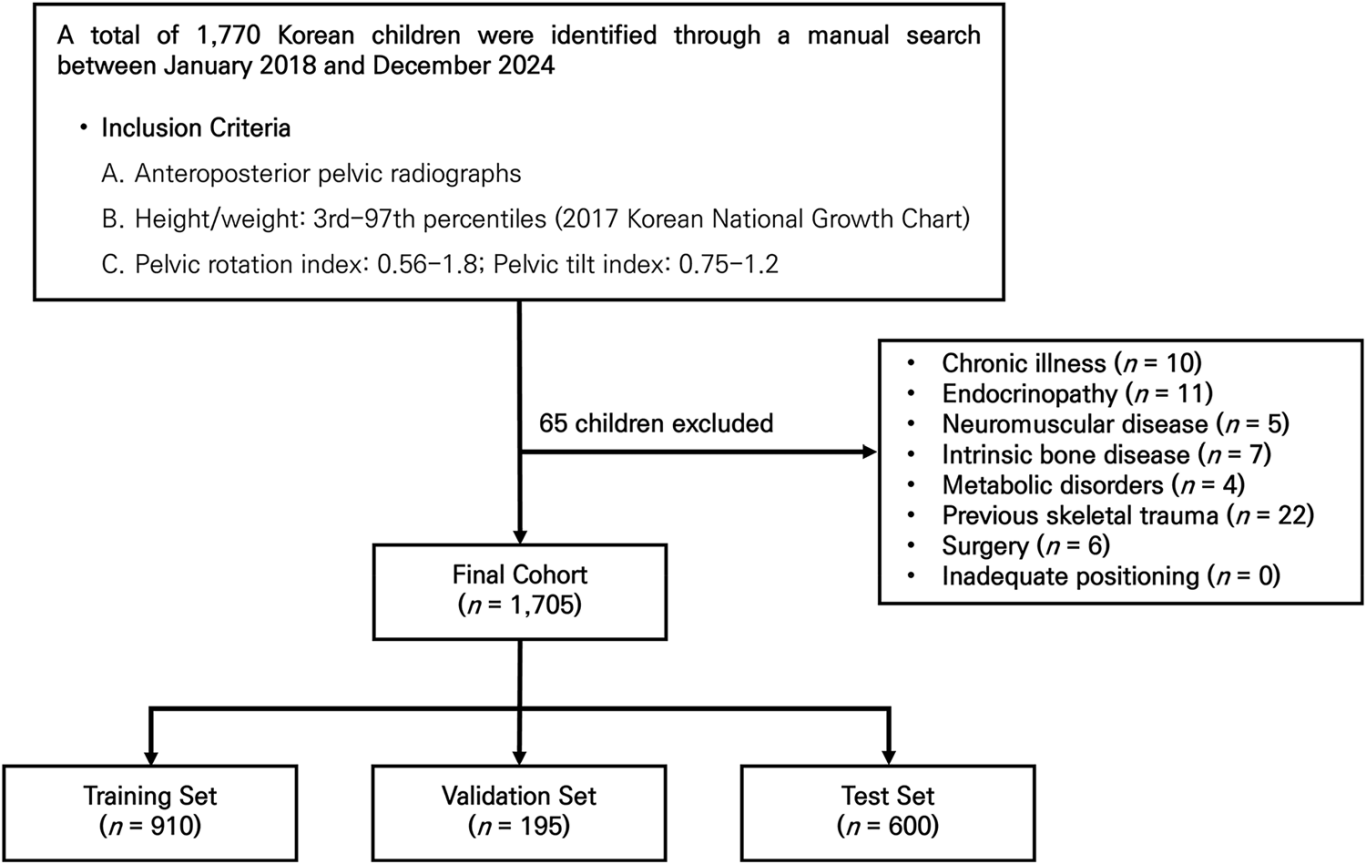
Flowchart of participant selection

From an initial pool of 1770 AP pelvic radiographs identified through PACS review, 65 cases were excluded based on predefined inclusion and exclusion criteria, resulting in a final cohort of 1705 healthy Korean children (Fig. 2). Of these, 910 children were allocated to the training set (mean age ± standard deviation [SD]: 5.18 ± 3.48 years; age range: 0.5–12 years; 441 male, 469 female), 195 children to the validation set (mean age ± SD: 5.16 ± 3.47 years; age range: 0.5–12 years; 108 male, 87 female), and 600 children to the test set (mean age ± SD: 5.23 ± 3.48 years; age range: 0.5–11 years; 315 male, 285 female). Participant characteristics are summarized in Table 1.

**Table 1:** Participant characteristics

Variable	Training set	Validation set	Test set
No. of participants	910	195	600
Age (years)	5.18 ± 3.48	5.16 ± 3.47	5.23 ± 3.48
Age range (years)	0.5–12	0.5–12	0.5–11
Sex			
Male (*n*)	441	108	315
Female (*n*)	469	87	285
FHOC size (mm)			25.25 ± 11.10
Height (cm)			102.57 ± 30.34

Except where otherwise indicated, data are expressed as mean ± standard deviation

*FHOC* femoral head ossification center

### Stage-wise validation performance

The three-stage cascaded DL pipeline demonstrated consistent performance across all stages with quantitative results summarized in Table 2. Representative segmentation results stratified by age group and sex are provided in Supplementary Figs. 1 and 2.

**Table 2:** Stage-wise validation performance of the three-stage cascaded DL algorithm for FHOC size measurement

Object detection	**Side**	**ROI class**	**mAP 0.5**			**mAP 0.5:0.95**		
	Left	Proximal femur	0.995			0.916		
	Right	Proximal femur	0.995			0.919		
	Average		0.995			0.918		
Segmentation	**Side**	**Segmentation masks**	**DSC**			**HD (mm)**		
	Left	FHOC	0.938			8.725		
	Right	FHOC	0.941			8.782		
	Average		0.940			8.754		
Landmark detection	**Side**	**Landmark**	**MRE (mm)**	**SDR (%)**				
				≤ 1 mm	≤ 2 mm	≤ 2.5 mm	≤ 3 mm	≤ 4 mm
	Left	The medial margin of the femoral head	0.154	98.7	98.8	98.8	99.0	99.2
		The outermost lateral point of the femoral head	0.131	99.0	99.2	99.2	99.2	99.3
	Right	The medial margin of the femoral head	0.161	98.5	98.8	99.2	99.3	99.3
		The outermost lateral point of the femoral head	0.152	98.0	98.2	98.3	98.3	98.3
	Average		0.150	98.75	98.75	98.88	98.95	99.03

*DSC* Dice similarity coefficient, *FHOC* femoral head ossification center, *HD* Hausdorff distance, *mAP* mean average precision, *MRE* mean radial error, *ROI* region of interest, *SDR* successful detection rates

### Automatic measurements of FHOC size

Agreement analysis demonstrated strong correlations between AI-derived and radiologist measurements of FHOC size (female: CCC = 0.996, 95% confidence interval (CI): 0.995–0.997; *r* = 0.996, 95% CI: 0.996–0.997; MAE = 0.785 mm, 95% CI: 0.742–0.830; RMSE = 0.950 mm, 95% CI: 0.885–1.025; male: CCC = 0.997, 95% CI: 0.996–0.997; *r* = 0.996, 95% CI: 0.996–0.997; MAE = 0.792 mm, 95% CI: 0.751–0.836; RMSE = 0.963 mm, 95% CI: 0.890–1.046). Bland–Altman analysis showed minimal mean bias (female: –0.02 mm; male: –0.05 mm) and narrow 95% LoA (female: –1.89 to –1.89 mm; male: –1.93 to +1.83 mm), with no evidence of proportional bias or heteroscedasticity, indicating that AI-derived and radiologist measurements are clinically interchangeable.

To further evaluate consistency across developmental stages, Bland–Altman plots were stratified by 12 predefined age subgroups for each sex. Mean differences were consistently small across both sexes (females: –0.43 to +0.20 mm; males: –0.23 to +0.18 mm), with LoA spanning –3.24 to +2.38 mm and –0.20 to +2.14 mm, respectively, across age groups. Across all subgroups, over 95% of all paired measurements fell within the 95% LoA across all age groups, and no systematic trend in bias was observed with increasing age.

**Fig. 3:**
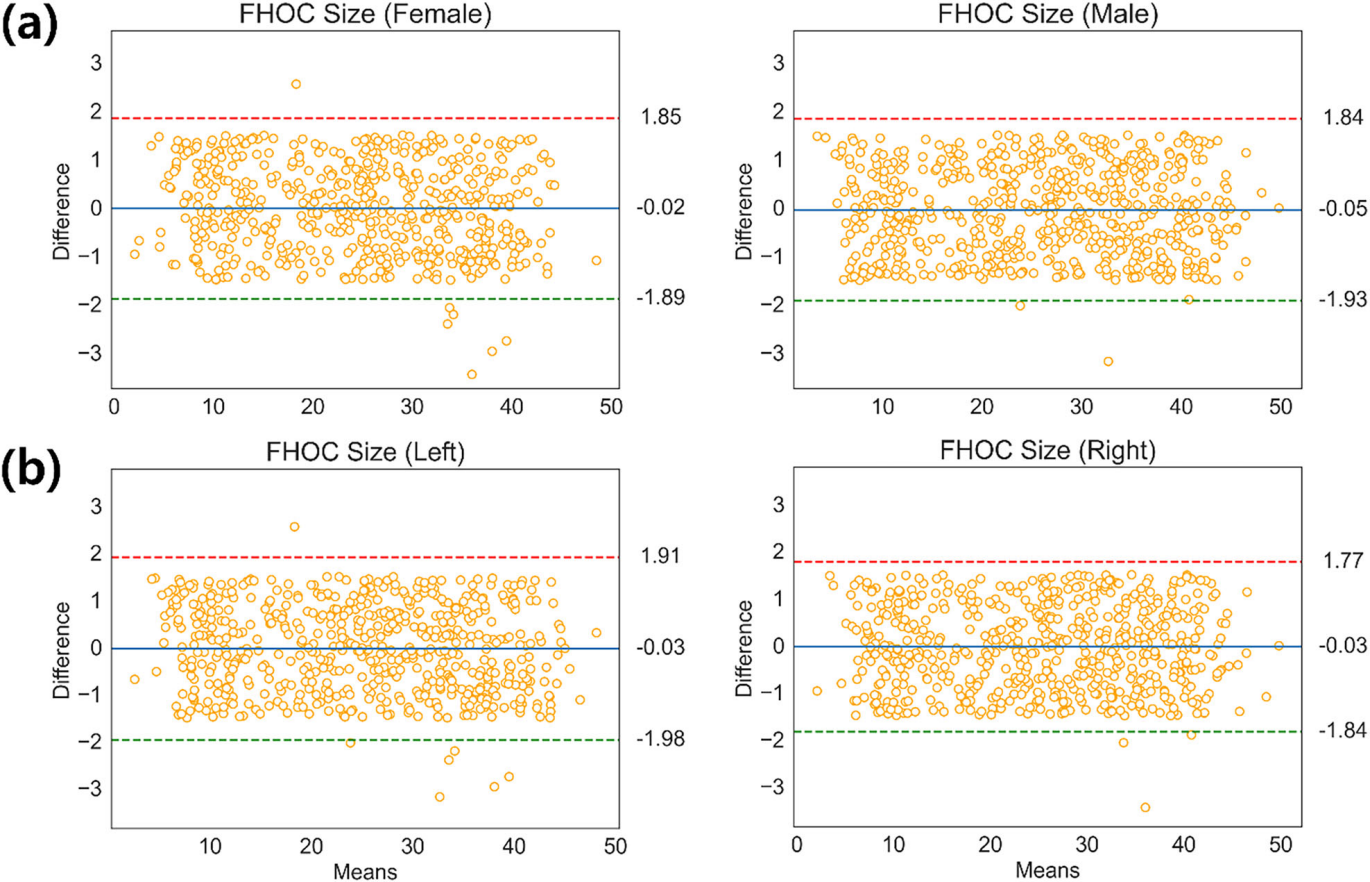
Bland–Altman plots comparing the reference standard and AI-derived FHOC size measurements in the entire test set stratified by sex and laterality. The *x*-axis represents the mean of both measurements (mm), while the *y*-axis represents their difference (mm). The blue dotted line indicates the mean difference (bias) between the two measurements. The red and green dotted lines denote the 95% upper and lower limits of agreement, respectively. **a** Stratified by sex. **b** Stratified by laterality. FHOC, femoral head ossification center

As a supplementary analysis, paired *t*-tests revealed no statistically significant mean differences between AI-derived and radiologist measurements (*p* > 0.05), supporting the observed agreement. However, given the growth-related variability of FHOC size, reliability was primarily evaluated using agreement metrics rather than hypothesis testing. Laterality-based analysis showed comparable agreement between left and right hips across all age groups. Detailed results are provided in Table 3, Fig. 3, and Supplementary Figs. 3–6.

**Table 3:** Correlation between FHOC size measurements using the reference standard and the AI-driven method across sex and laterality

Subgroups		Mean FHOC size [mean ± standard deviation] (mm)		*r*	CCC	MAE (mm)	RMSE (mm)	Paired *t*-tests
		Reference standard	Automatic measurement					
Sex	Female	24.524 ± 10.932	24.504 ± 10.794	0.996 (0.996, 0.997)*, p* < 0.001	0.996 (0.995, 0.997)	0.785 (0.742, 0.830)	0.950 (0.885, 1.025)	*p* = 0.622
	Male	25.917 ± 11.295	25.867 ± 11.293	0.996 (0.996, 0.997), *p* < 0.001	0.997 (0.996, 0.997)	0.792 (0.751, 0.836)	0.963 (0.890, 1.046)	*p* = 0.191
Laterality	Left	25.144 ± 11.118	25.107 ± 10.998	0.996 (0.995, 0.997), *p* < 0.001	0.996 (0.995, 0.997)	0.809 (0.765, 0.857)	0.991 (0.913, 1.076)	*p* = 0.368
	Right	25.367 ± 11.173	25.332 ± 11.160	0.996 (0.996, 0.997)*, p* < 0.001	0.996 (0.995, 0.997)	0.785 (0.742, 0.830)	0.950 (0.885, 1.025)	*p* = 0.353

Data in parentheses indicate 95% CIs

*FHOC* femoral head ossification center, *CCC* concordance correlation coefficient, *MAE* mean absolute error, *r* Pearson correlation coefficient, *RMSE* root mean square error

### Predictive modeling of FHOC size using AI-derived growth curves

The FHOC size exhibited a strong positive correlation with chronological age in both female (*r* = 0.955, *p* < 0.001) and male (*r* = 0.960, *p* < 0.001) children. Statistical comparisons revealed no significant differences between male and female children in the correlation between chronological age and FHOC size (*Z* = –0.284, *p* = 0.777), suggesting a similar age-related growth pattern across sexes. Similarly, left and right FHOC showed nearly identical correlations with age (left: *r* = 0.957, *p* < 0.001; right: *r* = 0.958, *p* < 0.001), with no significant laterality differences (*Z* = –1.309, *p* = 0.191), suggesting a consistent growth trajectory across sides.

**Fig. 4:**
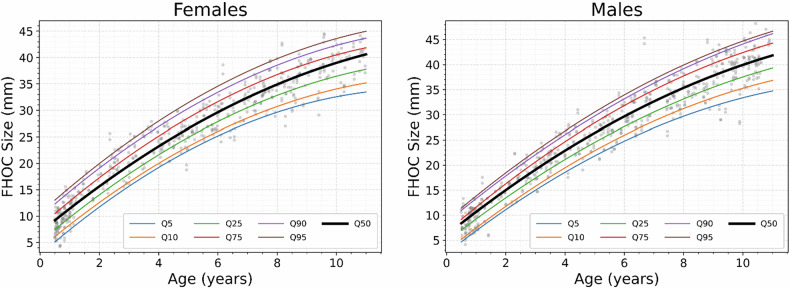
Quantile regression growth curves for FHOC size in healthy Korean children based on AI-derived measurements. The fitted percentile curves (5th–95th) are overlaid with raw FHOC size measurements (gray dots), illustrating their alignment with the observed data distribution across age and sex. FHOC, femoral head ossification center

Figure 4 depicts quantile regression-derived growth curves for female and male FHOC size. Reference ranges (5th, 50th, and 95th percentiles) for female and male FHOC sizes, derived from these quantile regressions, are presented in Table 4. Model performance demonstrated high explanatory power, with adjusted *R*² of 0.927 in females and 0.934 in males. Prediction accuracy was further evaluated across 12 age groups, showing consistently low MAE and RMSE (Table 5). These results support the model’s robustness and its potential utility in clinical assessment of skeletal maturation.

**Table 4:** Percentile values for FHOC sizes in healthy Korean children (0.5–11 years) based on regression analysis of AI-derived measurements on pediatric pelvic radiographs

Age (years)	Female			Male		
	5th Percentile	50th Percentile	95th Percentile	5th Percentile	50th Percentile	95th Percentile
0.5	4.664	8.386	11.418	5.097	9.202	13.031
1	6.887	10.662	13.836	7.397	11.420	15.425
2	11.095	15.009	18.450	11.713	15.640	19.952
3	14.987	19.082	22.768	15.649	19.571	24.130
4	18.562	22.883	26.790	19.204	23.212	27.959
5	21.821	26.410	30.516	22.379	26.565	31.438
6	24.764	29.664	33.946	25.174	29.628	34.567
7	27.390	32.644	37.079	27.589	32.402	37.347
8	29.701	35.352	39.917	29.624	34.887	39.778
9	31.694	37.786	42.458	31.278	37.083	41.859
10	33.372	39.947	44.703	32.552	38.990	43.591
11	34.733	41.835	46.652	33.446	40.608	44.974

*FHOC* femoral head ossification center

**Table 5:** Prediction errors of FHOC size across age groups using a regression-based growth curve model

Age (years)	Female		Male	
	MAE (mm)	RMSE (mm)	MAE (mm)	RMSE (mm)
0.5–0.75	2.110 (1.706, 2.531)	2.697 (2.175, 3.173)	1.986 (1.488, 2.513)	2.562 (1.979, 3.119)
0.75–1	2.239 (1.797, 2.725)	2.707 (2.174, 3.217)	1.599 (1.294, 1.910)	1.995 (1.642, 2.313)
1–2	1.768 (1.385, 2.163)	2.284 (1.862, 2.699)	1.887 (1.490, 2.305)	2.376 (1.884, 2.820)
2–3	2.530 (2.014, 3.117)	3.202 (2.553, 3.868)	2.280 (1.866, 2.708)	2.735 (2.305, 3.139)
3–4	2.976 (2.409, 3.514)	3.432 (2.855, 3.945)	1.838 (1.490, 2.205)	2.387 (1.996, 2.742)
4–5	2.056 (1.623, 2.468)	2.629 (2.101, 3.098)	2.511 (1.953, 3.069)	3.153 (2.577, 3.666)
5–6	2.121 (1.786, 2.452)	2.420 (2.117, 2.712)	2.372 (1.900, 2.846)	2.948 (2.493, 3.360)
6–7	2.758 (2.117, 3.471)	3.543 (2.701, 4.349)	2.434 (1.859, 3.131)	3.463 (2.257, 4.662)
7–8	2.313 (1.858, 2.790)	2.877 (2.368, 3.381)	2.735 (2.254, 3.255)	3.314 (2.727, 3.915)
8–9	2.290 (1.769, 2.834)	3.064 (2.394, 3.664)	2.823 (2.307, 3.407)	3.326 (2.731, 3.963)
9–10	2.492 (1.765, 3.275)	3.418 (2.412, 4.352)	3.014 (2.357, 3.725)	4.096 (3.215, 4.938)
10–11	2.486 (1.898, 3.126)	3.081 (2.391, 3.787)	2.625 (2.173, 3.113)	3.260 (2.734, 3.774)

Data in parentheses indicate 95% CIs

*FHOC* femoral head ossification center, *MAE* mean absolute error, *RMSE* root mean square error

## Discussion

In this study, we developed an AI-driven approach for FHOC size measurement and growth curve generation, thereby providing an objective and reproducible reference framework for pediatric hip joint assessment. The AI-derived measurements demonstrated excellent agreement with radiologist measurements, consistent across sex and developmental stages. These results indicate that the automated system may provide a robust and clinically reliable alternative to manual measurement.

Wegener et al [23] established reference values for the maximum capital femoral epiphysis diameter using manual measurements from supine pelvic radiographs. While both studies confirm a strong positive correlation between age and FHOC size, their reported FHOC values were systematically larger than those observed in our study population. This discrepancy likely arises from differences in population demographics, as Wegener et al [23] primarily included European children, whereas our study focused exclusively on Korean children. Additionally, manual measurement methods, as used in Wegener et al [23], are inherently subject to inter-observer variability, potentially contributing to measurement inconsistencies. In contrast, our AI-driven approach enables fully automated, standardized FHOC quantification, providing consistent and operator-independent measurements. While manual assessment by experienced radiologists remains the clinical standard, the AI system substantially reduces potential sources of intra- and inter-observer variability and may offer a scalable solution for longitudinal monitoring and multi-center studies. Moreover, our study advances beyond traditional normative growth assessments by introducing AI-derived growth curves and predictive modeling, providing an objective framework for early diagnosis and longitudinal evaluation of pediatric hip joint maturation. These enhancements position our methodology as a robust and clinically applicable tool for improving pediatric hip joint assessment in Asian populations.

In addition to its role in assessing normal hip joint development, the FHOC growth chart may offer supplementary value in specific pediatric hip joint disorders. One representative example is LCP disease, which results from an interruption of the blood supply to the femoral head. Although its exact etiology remains unclear, an association with recurrent transient synovitis has been suggested. However, differentiation between these two conditions on plain radiographs alone is often difficult [32]. Applying the FHOC growth chart, a smaller-than-expected FHOC size may serve as an indirect indicator for further evaluation, such as MRI or bone scintigraphy, thereby enhancing the likelihood of early detection of LCP. Another example is DDH. In patients treated with closed reduction, previous studies have demonstrated a negative correlation between femoral head diameter and the occurrence of avascular necrosis (AVN) of the proximal femoral epiphysis [33]. Accordingly, applying the FHOC growth chart to identify DDH patients with abnormally small FHOC size may provide a useful surrogate marker for predicting AVN risk during follow-up.

This study has some limitations. First, the model was developed and validated using retrospective single-center data from Korean children, which may limit generalizability across different ethnicities, institutions, and imaging conditions and protocols. External validation using multi-ethnic, multi-center cohorts—ideally incorporating variations in scanner types, image quality, and acquisition protocols—will be essential to confirm robustness and broader applicability across diverse clinical settings. Second, all reference FHOC size measurements were performed by a single board-certified radiologist to ensure consistency. However, intra- and inter-observer agreement was not assessed, and the model was not directly compared with additional human readers or conventional clinical measurement methods. Future studies should include multiple annotators and compare model performance against a panel of expert radiologists to quantify inter-rater reliability and more comprehensively evaluate clinical equivalence and added value. Third, the analysis relied on 2D radiographic images, which cannot fully represent the complex 3D morphology of the femoral head. While anteroposterior radiographs are the standard imaging modality in clinical pediatric hip assessments, future work may explore integrating 3D imaging modalities to better characterize morphometric features and validate the accuracy of 2D-derived measurements. Finally, only chronological age was included as a predictive factor for FHOC size, without incorporating other potentially relevant parameters such as bone age, weight, or skeletal maturity indicators (e.g., Risser stage). Incorporating these additional parameters may enhance the predictive accuracy of growth models and allow for more individualized assessment of developmental trajectories.

In conclusion, we developed an AI-driven approach for automated FHOC size measurement and pediatric hip growth assessment. The model demonstrated high precision and reproducibility, providing a standardized method for quantifying FHOC size. These AI-derived growth curves may serve as a valuable reference for evaluating pediatric hip joint development and enhancing the objectivity and consistency of manual radiographic assessment. Nevertheless, FHOC size might be regarded as an exploratory parameter at present. Therefore, rigorous external validation in disease-specific, multi-center cohorts is warranted to determine whether this metric can reliably inform diagnosis, prognostication, or therapeutic decision-making.

## Supplementary information

ELECTRONIC SUPPLEMENTARY MATERIAL

## Abbreviations

AI: Artificial intelligence
AP: Anteriorposterior
CCC: Concordance correlation coefficient
CI: Confidence intervals
DL: Deep learning
FHOC: Femoral head ossification center
MAE: Mean absolute error
PACS: Picture archiving and communication system
*r*: Pearson correlation coefficient
RMSE: Root mean square error
