# Supplementary material for: Deep learning-based automatic measurement of the femoral head ossification center in healthy Korean children: development of a novel radiographic growth chart
Source: Eur Radiol. 2026 Jan 13;36(6):4989–99. doi: 10.1007/s00330-025-12263-z (PMC13212369; doi:10.1007/s00330-025-12263-z)
Supplement: Supplementary file 1 — ELECTRONIC SUPPLEMENTARY MATERIAL [file 330_2025_12263_MOESM1_ESM.pdf]

Deep learning-based automatic measurement of the femoral head ossification center in healthy Korean children: Development of a novel radiographic growth chart

Electronic Supplementary Material

Supplementary Figure 1. Representative segmentation results of FHOC across age groups in female participants. The left panel shows the input anteroposterior pelvic radiograph, the middle panel presents the ground-truth segmentation mask, and the right panel displays the predicted contour generated by the proposed deep learning model, demonstrating close agreement with the reference boundary.

Supplementary Figure 2. Representative segmentation results of FHOC across age groups in male participants. The left panel shows the input anteroposterior pelvic radiograph, the middle panel presents the ground-truth segmentation mask, and the right panel displays the predicted contour generated by the proposed deep learning model, demonstrating close agreement with the reference boundary.

Supplementary Figure 3. Bland–Altman plots comparing the reference standard and AI-derived FHOC size measurements, stratified by 12 age groups for female participants. For each plot, the x-axis represents the mean of the two measurements (mm), and the y-axis represents their difference (mm). The blue dotted line indicates the mean difference (bias), while the red and green dotted lines denote the 95% upper and lower limits of agreement, respectively. This stratified analysis accounts for the age-dependent variation in FHOC size and allows visualization of potential age-specific measurement bias.

Supplementary Figure 4. Bland–Altman plots comparing the reference standard and AI-derived FHOC size measurements, stratified by 12 age groups for male participants. For each plot, the x-axis represents the mean of the two measurements (mm), and the y-axis represents their difference (mm). The blue dotted line indicates the mean difference (bias), while the red and green dotted lines denote the 95% upper and lower limits of agreement, respectively. This stratified analysis accounts for the

age-dependent variation in FHOC size and allows visualization of potential age-specific measurement bias.

Supplementary Figure 5. Bland–Altman plots comparing the reference standard and AI-derived FHOC size measurements for the left hip, stratified by 12 age groups. For each plot, the x-axis represents the mean of the two measurements (mm), and the y-axis represents their difference (mm). The blue dotted line indicates the mean difference (bias), while the red and green dotted lines denote the 95% upper and lower limits of agreement, respectively. This stratified analysis accounts for the age-dependent variation in FHOC size and allows visualization of potential age-specific measurement bias.

Supplementary Figure 6. Bland–Altman plots comparing the reference standard and AI-derived FHOC size measurements for the right hip, stratified by 12 age groups. For each plot, the x-axis represents the mean of the two measurements (mm), and the y-axis represents their difference (mm). The blue dotted line indicates the mean difference (bias), while the red and green dotted lines denote the 95% upper and lower limits of agreement, respectively. This stratified analysis accounts for the age-dependent variation in FHOC size and allows visualization of potential age-specific measurement bias.

Supplementary Figure 7. Overview of the DualBranchFusion-Net architecture.

Supplementary Figure 8: Schematic illustration of the FHOC size measurement procedure.

Supplementary Appendix S1. Development of the object detection network

Supplementary Appendix S2. Development of the segmentation network

Supplementary Appendix S3. Development of the landmark detection algorithm

Supplementary Figure 1. Representative segmentation results of FHOC across age groups in female participants. The left panel shows the input anteroposterior pelvic radiograph, the middle panel presents the ground-truth segmentation mask, and the right panel displays the predicted contour generated by the proposed deep learning model, demonstrating close agreement with the reference boundary.

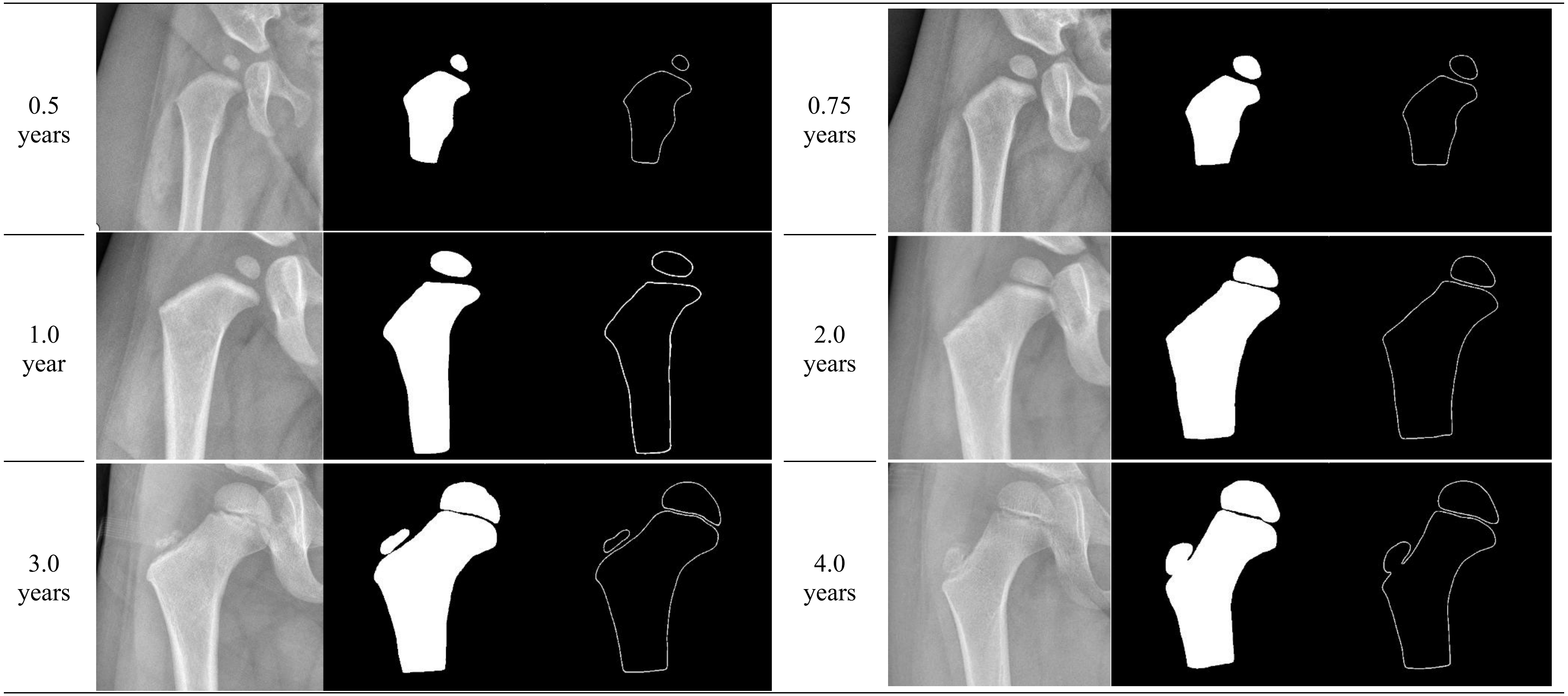

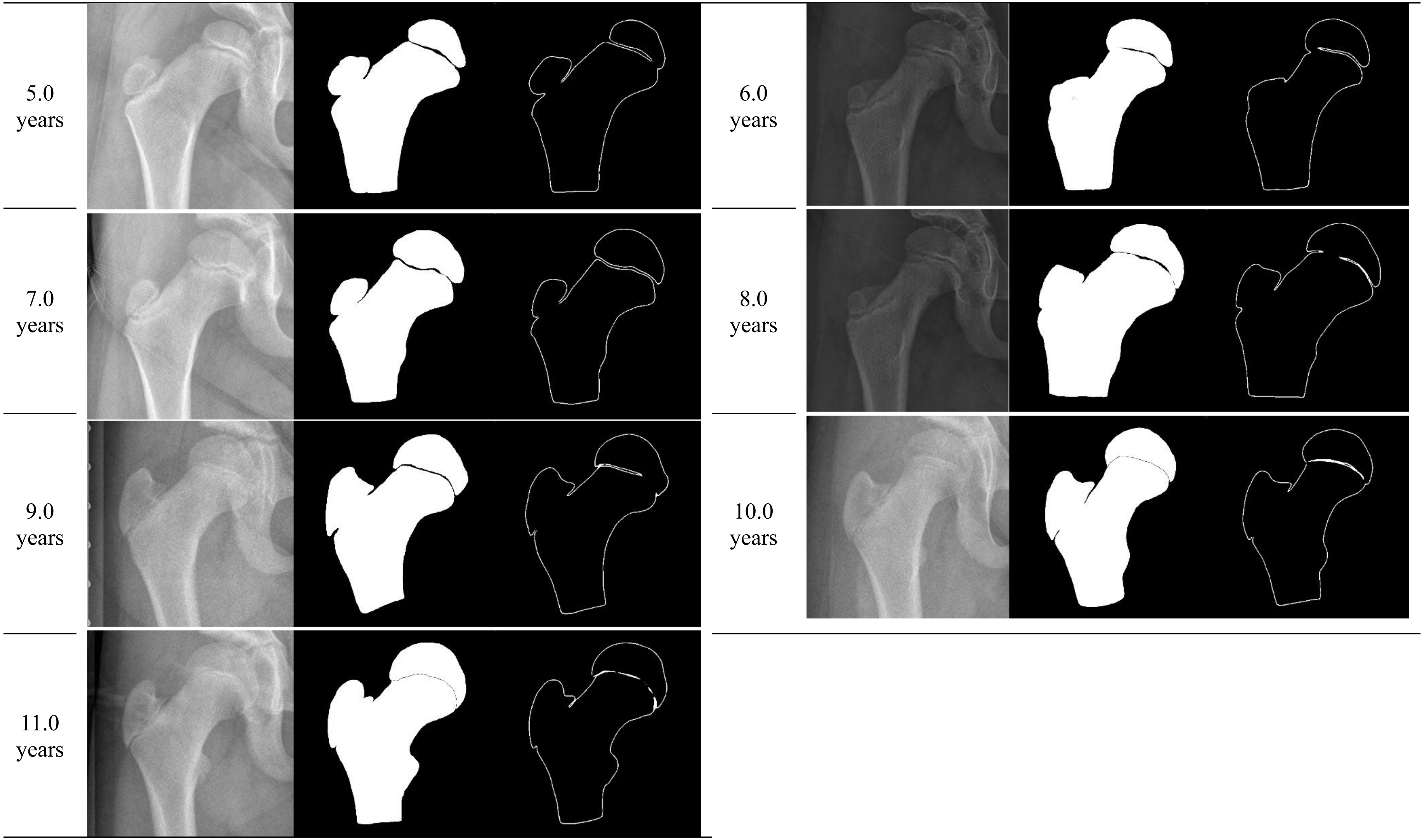

5.0 years
6.0 years
7.0 years
8.0 years
9.0 years
10.0 years
11.0 years

Supplementary Figure 2. Representative segmentation results of FHOC across age groups in male participants. The left panel shows the input anteroposterior pelvic radiograph, the middle panel presents the ground-truth segmentation mask, and the right panel displays the predicted contour generated by the proposed deep learning model, demonstrating close agreement with the reference boundary.

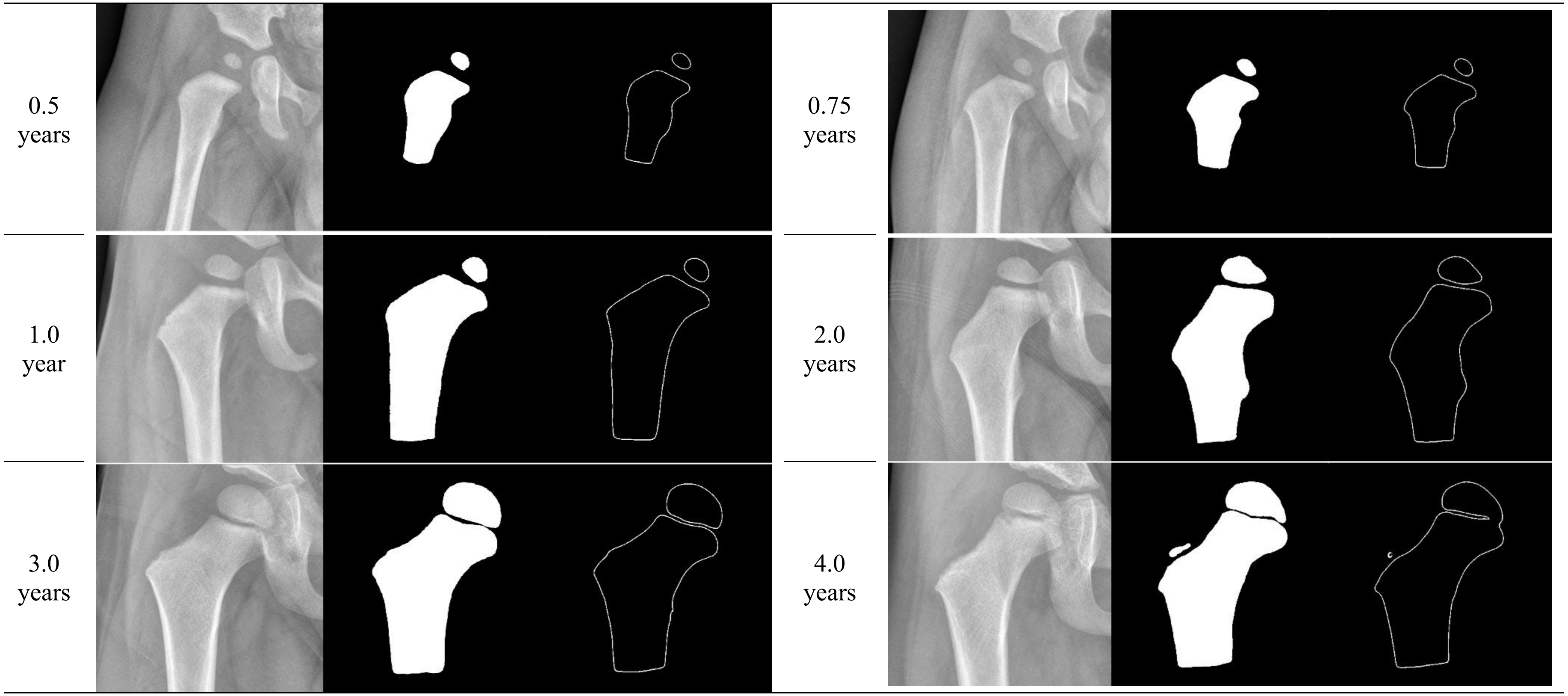

5.0 years	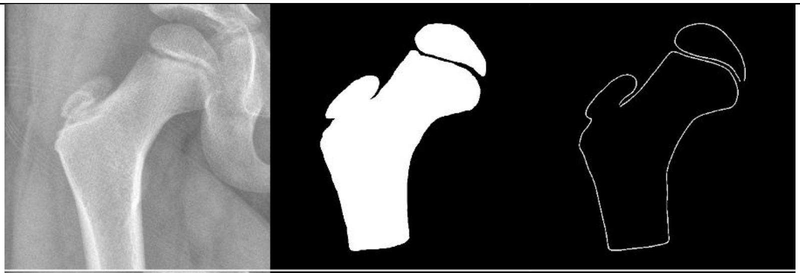	6.0 years	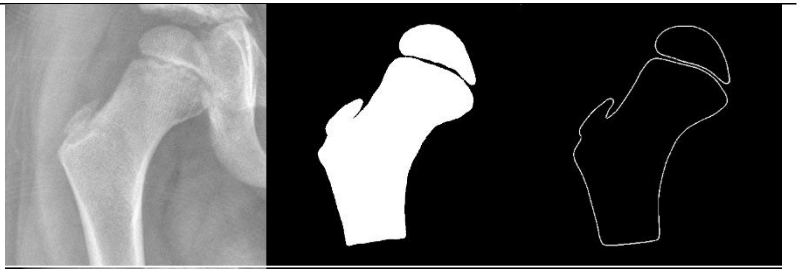
7.0 years	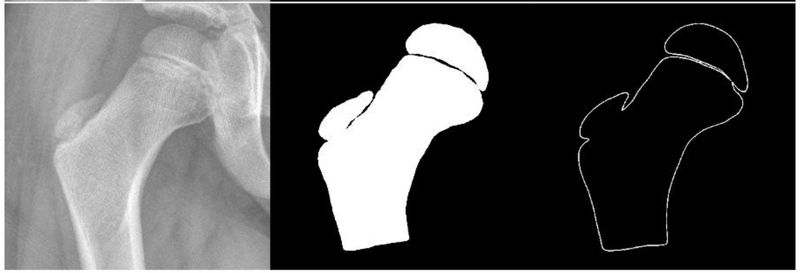	8.0 years	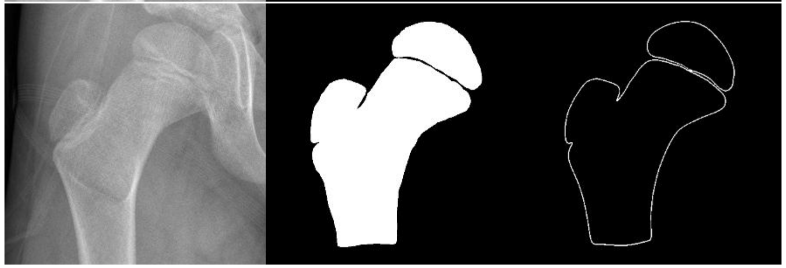
9.0 years	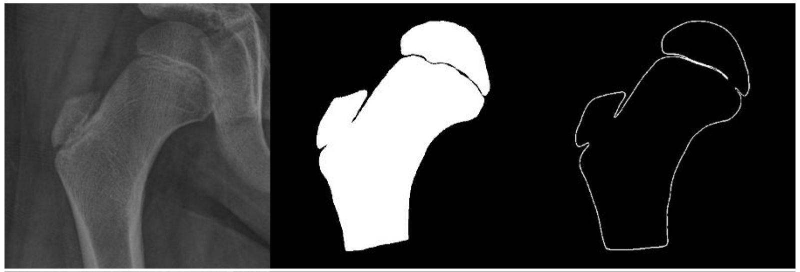	10.0 years	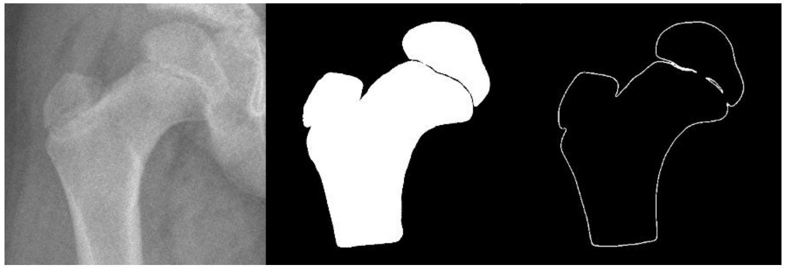
11.0 years	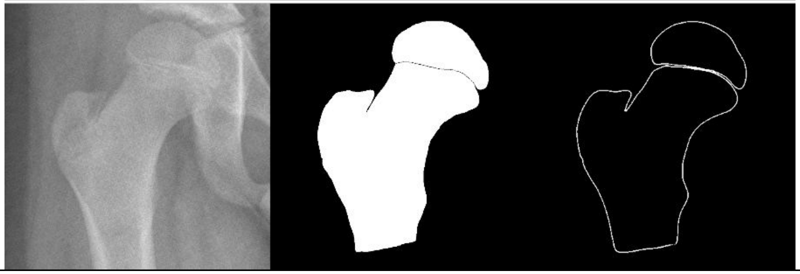		

Supplementary Figure 3. Bland–Altman plots comparing the reference standard and AI-derived FHOC size measurements, stratified by 12 age groups for female participants. For each plot, the x-axis represents the mean of the two measurements (mm), and the y-axis represents their difference (mm). The blue dotted line indicates the mean difference (bias), while the red and green dotted lines denote the 95% upper and lower limits of agreement, respectively. This stratified analysis accounts for the age-dependent variation in FHOC size and allows visualization of potential age-specific measurement bias.

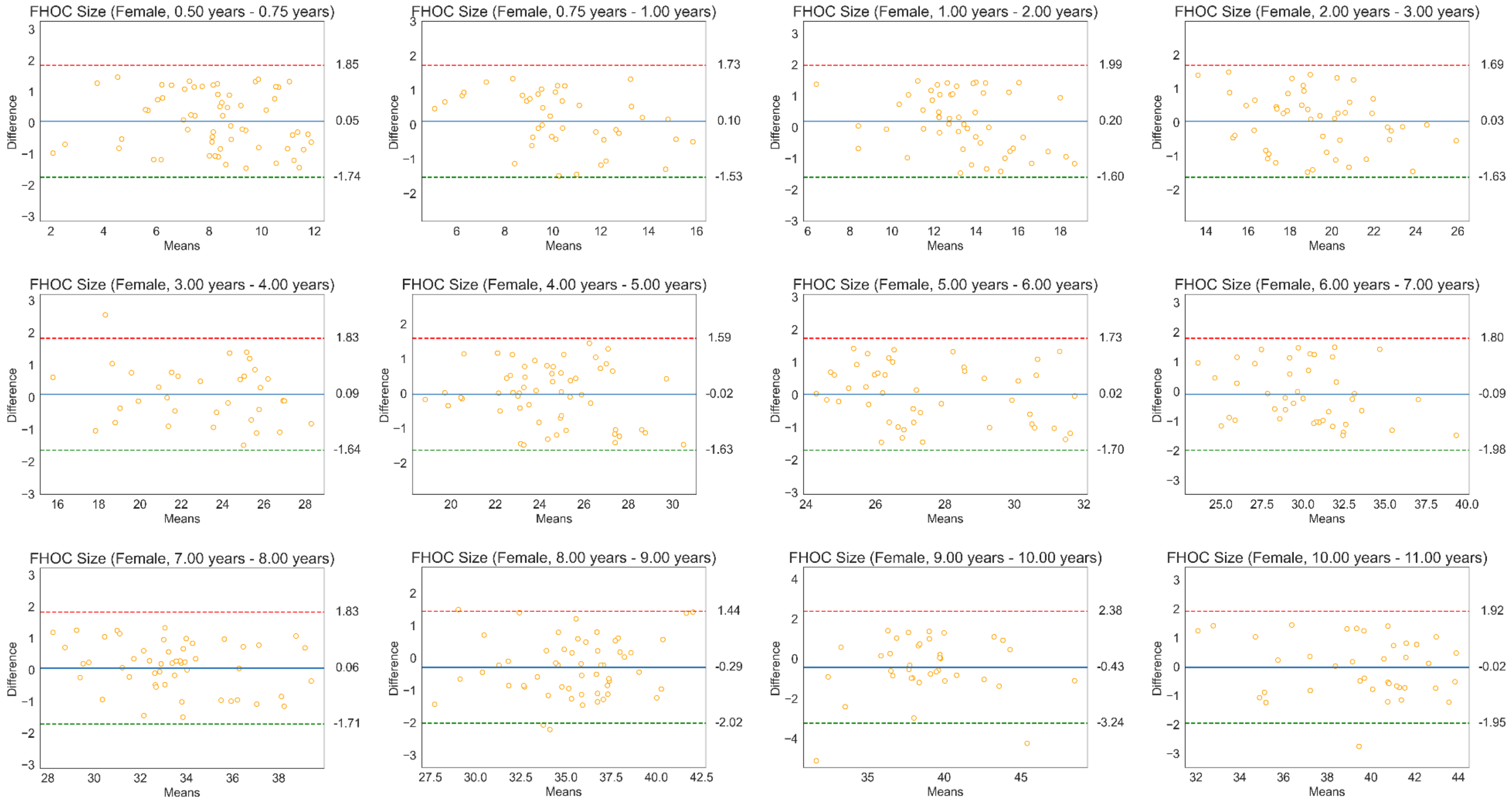

Supplementary Figure 4. Bland–Altman plots comparing the reference standard and AI-derived FHOC size measurements, stratified by 12 age groups for male participants. For each plot, the x-axis represents the mean of the two measurements (mm), and the y-axis represents their difference (mm). The blue dotted line indicates the mean difference (bias), while the red and green dotted lines denote the 95% upper and lower limits of agreement, respectively. This stratified analysis accounts for the age-dependent variation in FHOC size and allows visualization of potential age-specific measurement bias.

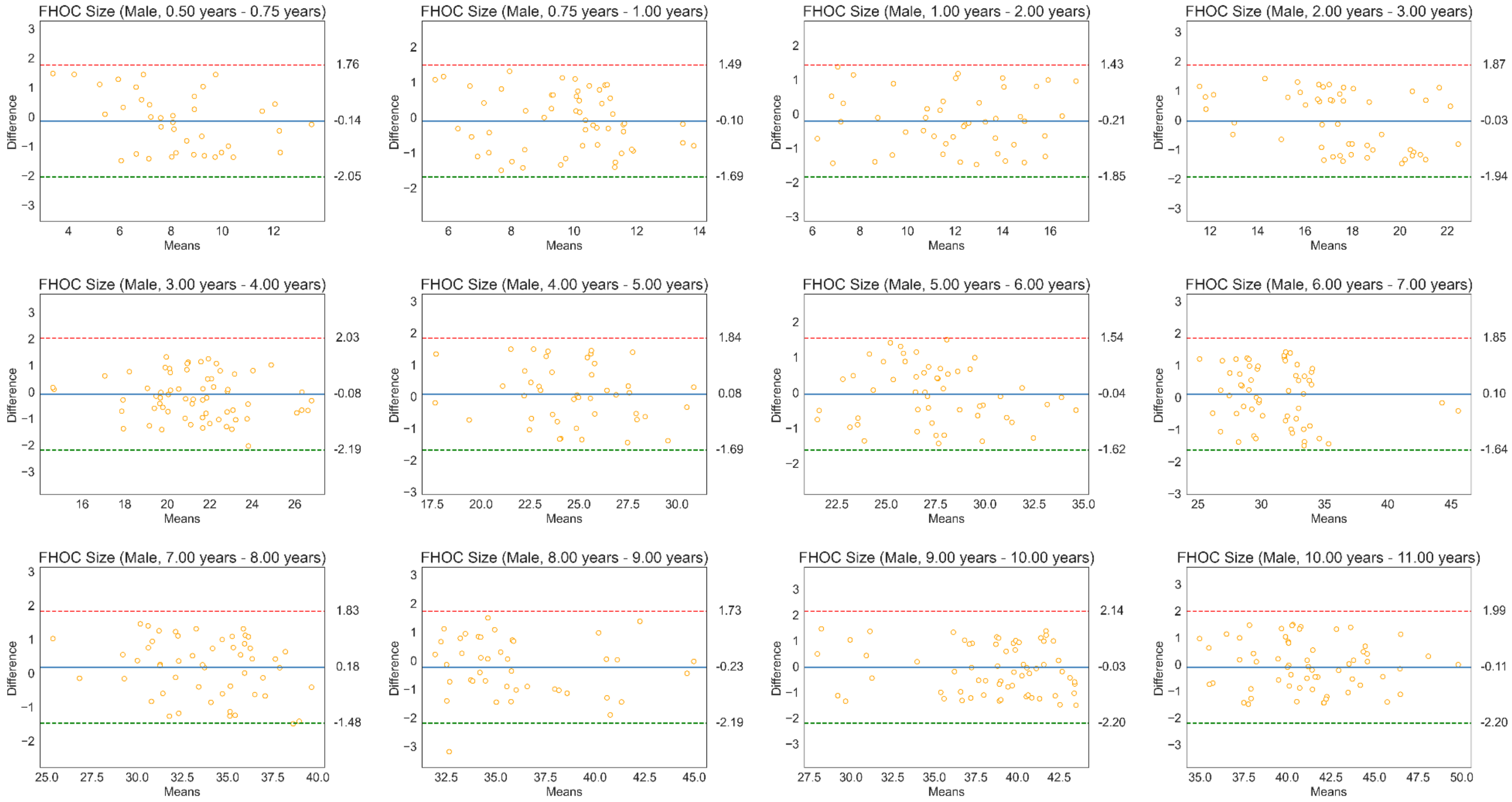

Supplementary Figure 5. Bland–Altman plots comparing the reference standard and AI-derived FHOC size measurements for the left hip, stratified by 12 age groups. For each plot, the x-axis represents the mean of the two measurements (mm), and the y-axis represents their difference (mm). The blue dotted line indicates the mean difference (bias), while the red and green dotted lines denote the 95% upper and lower limits of agreement, respectively. This stratified analysis accounts for the age-dependent variation in FHOC size and allows visualization of potential age-specific measurement bias.

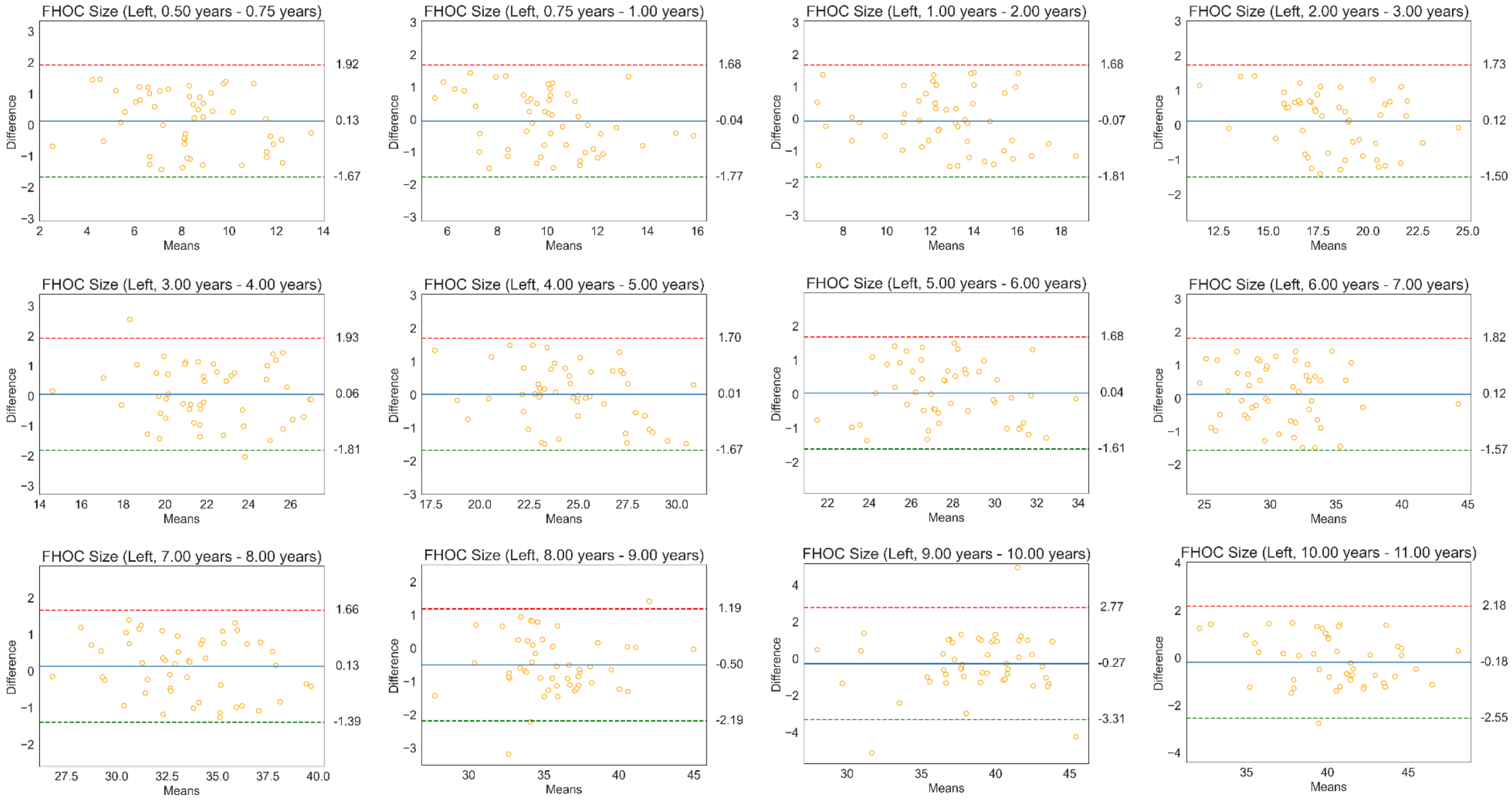

Supplementary Figure 6. Bland–Altman plots comparing the reference standard and AI-derived FHOC size measurements for the right hip, stratified by 12 age groups. For each plot, the x-axis represents the mean of the two measurements (mm), and the y-axis represents their difference (mm). The blue dotted line indicates the mean difference (bias), while the red and green dotted lines denote the 95% upper and lower limits of agreement, respectively. This stratified analysis accounts for the age-dependent variation in FHOC size and allows visualization of potential age-specific measurement bias.

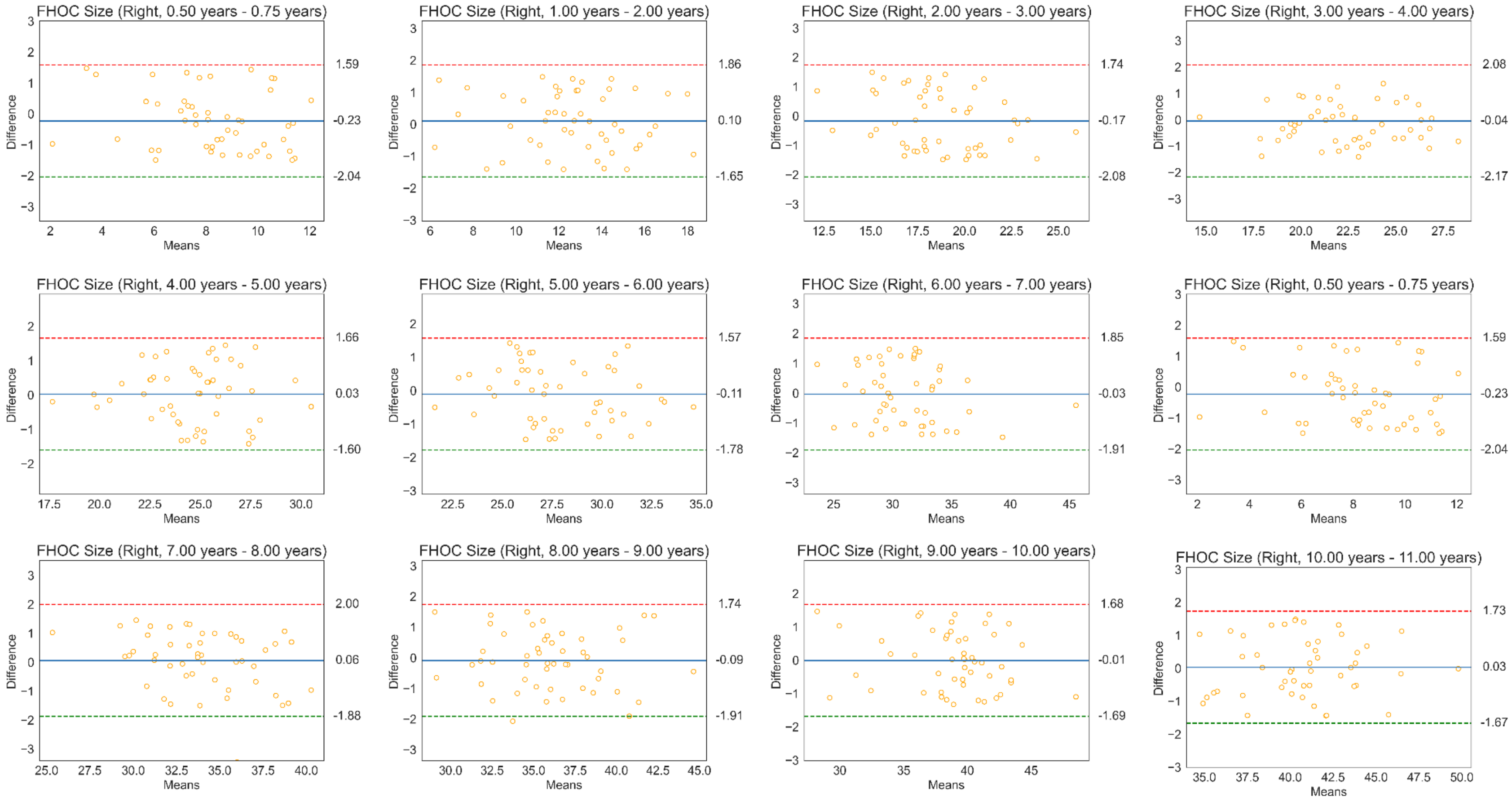

Supplementary Appendix 1. Development of the object detection network

Deep learning algorithm

We adopted YOLOv8x [1] to detect proximal femurs bilaterally. YOLOv8x offers high detection precision at higher resolutions while maintaining a relatively lower computational cost, making it well-suited for processing large-scale medical imaging data.

Data preprocessing

To address variations in image intensity across the dataset, image normalization was applied, scaling pixel values between 0 and 1 to reduce source-dependent variance and enhance contrast levels. The input images were then resized to 640 × 640 for both training and testing. In cases where the image height exceeded the width, zero-padding was applied to the left and right sides to ensure a square aspect ratio before resizing.

Initially, conventional data augmentation techniques, such as affine transformations (e.g., shifting, flipping, and zooming), were explored. However, as these did not improve performance, no data augmentation was applied in the final preprocessing pipeline.

Training details

The entire model was trained end-to-end using the stochastic gradient descent optimizer with a mini-batch size of 16, and the total number of training epochs was set to 500. Model performance was monitored after each epoch using the validation set, and early stopping was employed if no improvement was observed for 50 consecutive epochs following the epoch with the highest validation performance. The learning rate was set to 0.01. The deep learning models were implemented using the PyTorch framework (version 1.13.0) with a CUDA backend (version 11.8) and the cuDNN library (version 8.2). All training and testing were performed on a single NVIDIA A100 GPU with 80 GB RAM (NVIDIA, Santa Clara, CA, USA).

Supplementary Appendix 2. Development of the segmentation network

Deep learning algorithm

As epiphyseal fusion begins, the boundary between the femoral head ossification center (FHOC) and the proximal femoral metaphysis becomes indistinct, posing challenges for precise segmentation. To address this, we developed DualBranchFusion-Net, an encoder-decoder–based segmentation network designed to accurately delineate FHOC boundaries even in the presence of fusion-related morphological changes (Supplementary Figure 7).

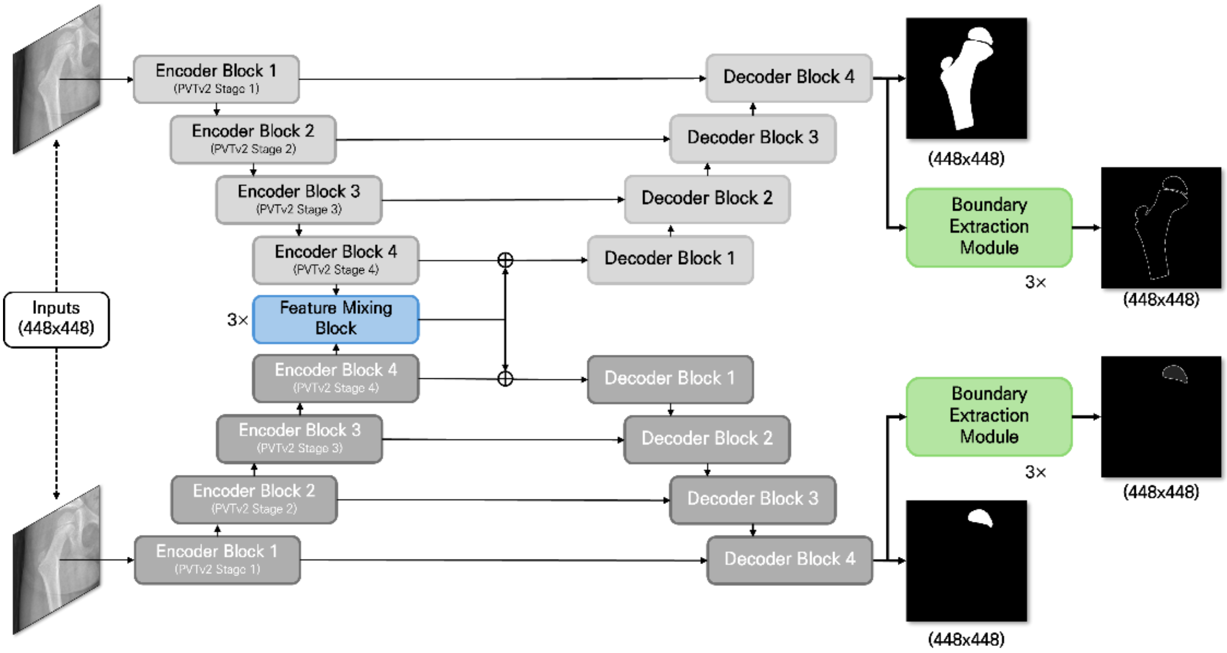

Supplementary Figure 7. Overview of the DualBranchFusion-Net architecture.

The DualBranchFusion-Net is designed to enhance FHOC boundary delineation, particularly in cases of epiphyseal fusion. It features a dual-branch architecture, wherein each branch follows an encoder-decoder structure. One branch is specialized for segmenting the FHOC region, while the other extracts the entire proximal femur from the input image. By leveraging multi-task learning with these complementary branches, the network achieves more precise FHOC segmentation, effectively addressing boundary ambiguity and improving overall segmentation accuracy. Detailed information on the deep learning model architecture is available in the thesis by Moon [2].

Data preprocessing

For data augmentation, we applied a simple data augmentation scheme based on horizontal flip. The input images were resized to 448 × 448 for both training and testing. Additionally, as with the object detection and landmark detection network training, normalization was applied by dividing all pixel values by 255 to scale them between 0 and 1.

Training details

For model training, a total of 300 epochs were set with a batch size of 4. To ensure efficient training, performance was evaluated on the validation set after each epoch. As with the object detection network training, early stopping was applied, halting the training if no improvement was observed for 50 epochs after the best-performing epoch. The initial learning rate was set to 0.001, and the AdamW optimizer was employed to enhance both training speed and stability. Additionally, to prevent overfitting and dynamically adjust the learning rate, the ReduceLROnPlateau scheduler [3] was used.

Supplementary Appendix 3. Development of the landmark detection algorithm

For landmark detection, the algorithm first identifies the medial margin and the outermost lateral point of the femoral head, which are required for measuring the maximum transverse diameter of the capital femoral epiphysis. This procedure is performed using the mask generated by the FHOC segmentation network. For clarity of explanation, the right pelvis is assumed as a reference. The contour point set of the segmentation mask is defined as $C = \{(x_i, y_i)\}_{i=1}^{N}$. In addition, the image coordinate system is defined such that the x-axis increases from left to right, and the y-axis increases from top to bottom.

Step 1) For the contour point set C obtained from the segmented mask, the centroid P_{center} is computed.

Step 2) Within the contour point set, the points with the minimum and maximum x-coordinates are denoted as P_{xmin} and P_{xmax}, respectively.

Step 3) Along the contour from P_{xmin} to P_{xmax}, the set is divided into two segments by the vertical line passing through P_{center}. The left and right contour subsets are denoted as C_L and C_R, respectively. Within each subset, the points with the maximum y-coordinate are defined as P_L and P_R.

Step 4) The entire contour set C is rotated counterclockwise by an alignment angle θ_{align}, which is determined from the slope of the line connecting P_L and P_R, so that the line segment between P_L and P_R becomes horizontally aligned.

Step 5) In the aligned contour set, the points with the maximum and minimum xxx-coordinates are newly identified and denoted as P_{start} and P_{end}, respectively.

Step 6) Subsequently, all points are rotated clockwise by θ_{align} to restore the original orientation, and the length of the line segment connecting P_{start} and P_{end} is measured as the FHOC size.

The overall procedure is schematically illustrated in Supplementary Figure 8.

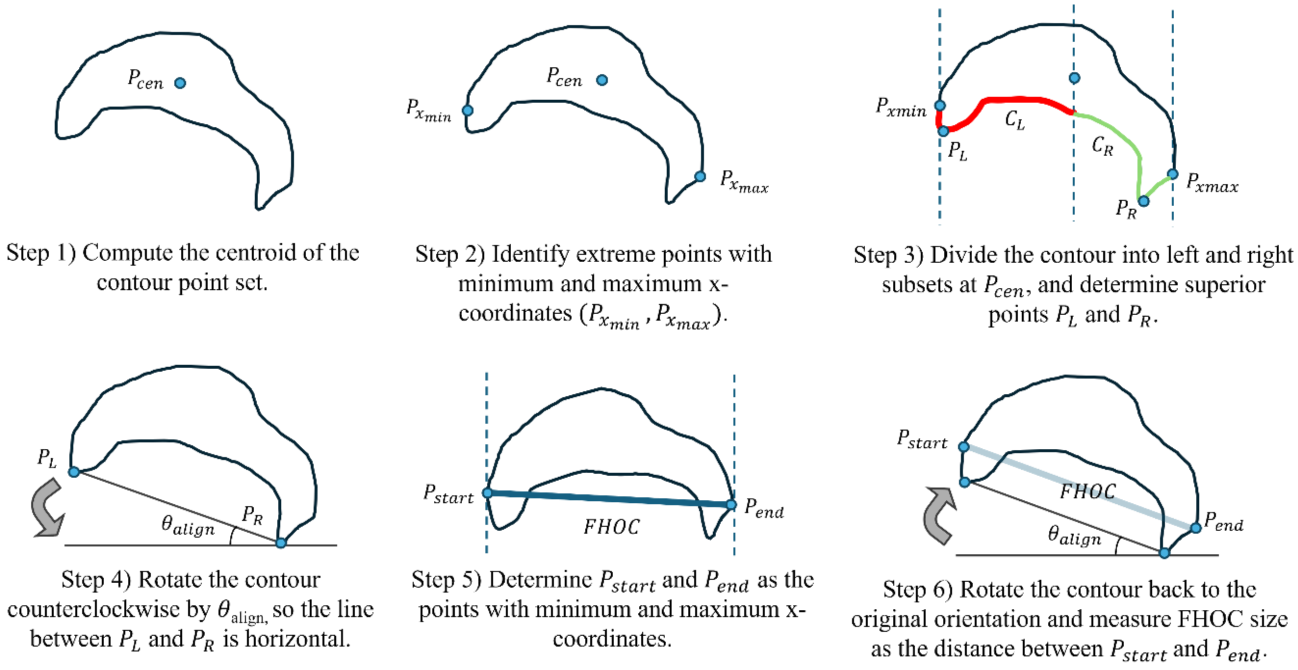

Supplementary Figure 8: Schematic illustration of the FHOC size measurement procedure.

References

[1] Jocher G, Chaurasia A, Qiu J. Ultralytics YOLO (2023). https://github.com/ultralytics/ultralytics?tab=readme-ov-file. Accessed April 14, 2025

[2] Moon KR (2025) Deep learning-based femur segmentation for enhanced growth analysis in pediatric development, Master's thesis. Kyonggi University, Republic of Korea.

[3] PyTorch: ReduceLROnPlateau—PyTorch 1.9.0 documentation. https://pytorch.org/docs/stable/generated/torch.optim.lr_scheduler.ReduceLROnPlateau.html#torch.optim.lr_scheduler.ReduceLROnPlateau. Accessed April 14, 2025
